# Temporal trends in and relationships between screen time, physical activity, overweight and obesity

**DOI:** 10.1186/1471-2458-12-1060

**Published:** 2012-12-08

**Authors:** Mitch J Duncan, Corneel Vandelanotte, Cristina Caperchione, Christine Hanley, W Kerry Mummery

**Affiliations:** 1 CQUniversity, Institute for Health and Social Science Research, Centre for Physical Activity Studies, Rockhampton, Bld 18, CQUniversity Australia, Rockhampton, QLD, 4702, Australia; 2 Faculty of Health and Social Development, University of British Columbia, Kelowna, Canada; 3 Faculty of Physical Education and Recreation, University of Alberta, Edmonton, Canada

**Keywords:** Temporal trends, Obesity, Screen time, Physical activity, Adults

## Abstract

**Background:**

The aims of this study were to examine temporal trends in the prevalence of sufficient moderate-to-vigorous intensity physical activity (MVPA), high levels of screen time, combined measures of these behaviors and overweight or obesity in Australian adults during the period 2002–2008. Trends over this time period in overweight or obesity within each behavior group (sufficient/insufficient MVPA, high/low screen time and combined behaviors) were also examined.

**Methods:**

Data were collected via annually conducted cross-sectional computer-assisted-telephone-interviews (CATI) of adults (n=7908) living in Central Queensland, Australia (2002–2008). Self-reported MVPA, screen time (TV viewing and computer use), and BMI were used to create dichotomous classifications of physical activity (Sufficient MVPA (S-MVPA), Insufficient Physical Activity (I-MVPA)), screen time (High Screen Time (HST), Low Screen Time (LST)), combined behavior categories (S-MVPA/LST, I-MVPA/LST, S-MVPA/HST, I-MVPA/HST) and BMI (Overweight or Obese, Healthy Weight) respectively.

**Results:**

The prevalence of S-MVPA, HST, and overweight or obesity increased at approximately the same rate over the study period in the overall sample and females (p≤0.05). In the overall sample and in females, the prevalence of overweight and obesity increased over the study period in those individuals classified as I-MVPA/HST (p≤0.05).

**Conclusion:**

Results provide evidence that while the prevalence of S-MVPA appears to be modestly increasing, the proportion of the population engaging in HST and classified as overweight or obese are increasing at approximately the same rate. These observations highlight the need to increase levels of total physical activity (including light intensity physical activity) and decrease sedentary behavior including screen time.

## Background

The health benefits of physical activity are numerous and well documented particularly in relation to individuals achieving sufficient levels of moderate-to-vigorous intensity physical activity (MVPA), or sufficient MVPA [1]. Sufficient MVPA is frequently defined as achieving 150 minutes of MVPA in 5 or more sessions in the previous week [2]. Though MVPA levels are generally low in the US, Australia and Canada, the population prevalence of individuals engaging in sufficient MVPA has gradually increased over recent years [3-6], and has occurred during periods when the prevalence of overweight or obesity has also increased [3,7-9]. Yet trends in the prevalence of sufficient MVPA and overweight or obesity are not equal across population groups, with significant differences observed by gender and age [5,9,10]. This apparent paradox in the trends of sufficient MVPA and overweight or obesity may due to reductions in levels of total physical activity due to decreased light intensity physical activity and increased sedentary behavior, increased energy intake or a combination of these behaviors [7,11].

Measures of screen based activities performed in seated postures, such as TV viewing and computer use, are commonly used indicators of sedentary behavior as the low metabolic cost of these activities is below the energy expenditure threshold used to define sedentary behavior (<1.5 METS) [12,13]. Time spent watching TV and using computers are screen based activities which are associated with increased risk of weight gain, overweight and obesity, diabetes, and CVD mortality [14-16]. Furthermore, the associations between increased screen based activity and the negative health outcomes remain present even when adjusted for engagement in MVPA. As such, the time spent engaged in sedentary behavior is emerging as a potentially important and independent risk factor for ill health [17,18].

There is little data on temporal trends in sedentary behaviors when compared to data on trends in MVPA. In occupational settings, physical activity has declined resulting in increased sedentary behavior in workplaces over recent decades [3,10,19]. In non-occupational settings sedentary behavior also appears to be increasing. In the US, time spent watching TV increased approximately 36 minutes every decade in the period 1950–2000 [3]; in Australia, average time spent in non-occupational sedentary behavior increased from 894 minutes/2 days in 1997 to 906 minutes/2 days in 2006 [20]. Furthermore comparisons in the trends of sufficient MVPA and engagement in sedentary behavior are infrequently performed within the same population [20], or the observed trends are difficult to interpret due to changes in survey instruments and methodologies [6,10]. Examining changes in both behaviors is useful in understanding how these two behaviors have changed over time in relation to disease outcomes and risk factors such as overweight or obesity.

Therefore the purpose of this study is threefold, firstly, to examine the relationships between screen time, MVPA and combined measures of these behaviors and being classified as overweight or obese in a pooled sample of Australian adults who completed separate cross sectional surveys in the period 2002–2008. Similar associations have been conducted previously [16,21], and are conducted in the current study to establish the associations between activity behaviors and overweight or obesity in the current sample prior to progressing to the other aims of the study. Secondly, the study aimed to examine temporal trends in the prevalence of these behaviors and being classified as overweight or obese during this period. Thirdly, the study sought to examine temporal trends in the prevalence of being classified as overweight or obese during this period within each of the behavior groups examined (sufficient/insufficient MVPA, high/low screen time and combined behaviors). These relationships will also be examined for the overall study population and within the male and female samples separately.

## Methods

### Sample

This study uses data pooled from a series of separate cross-sectional surveys of the adult population living in Central Queensland, Australia. The surveys were conducted annually by the Population Research Laboratory, at CQUniversity during the period 2002 to 2008. The samples sizes for each survey year are provided in Table 1, the overall sample size was 7908. Details on the survey methods are provided elsewhere and each survey was approved by CQUniversity’s Human Research Ethics Committee [5]. Briefly, each survey was performed during October and November using computer-assisted-telephone-interviewing (CATI), all respondents were aged 18 years or older (range 18–93 years), and were living in a dwelling contactable by direct-dialed land based telephone. Participants were randomly selected using a two-stage stratified sampling process where households (as phone numbers are linked to households) were selected first and then individuals within households were selected. Potential telephone numbers were drawn from commercially available Electronic White Pages, all duplicate, cellular and business numbers were removed from the sample before each survey commenced. Each year the sample was stratified by gender to reflect the characteristics of the Australian population based on the Census closest to the survey date, however the sample is not intended to be a representative sample of the Australian population. The response rate for the surveys ranged from 39.3% in 2007 to 62.3% in 2005 with an average response rate across surveys of 46.9%. Response rates are comparable to those reported in other recently conducted CATI based surveys [22,23], and no information is available on those who did not participate in the survey.

**Table 1:** Unweighted sample proportions of central queensland social survey participants by selected socio-demographic and behavioral categories 2002-2008

	**2002 (n=1127)**	**2003 (n=1147)**	**2004 (n=1102)**	**2005 (n=1127)**	**2006 (n=1131)**	**2007 (n=1112)**	**2008 (n=1162)**	**Overall Sample (n=7908)**
**Gender**								
Male	52.1	51.9	51.1	51.6	51.5	52.0	50.9	51.6
Female	47.9	48.1	48.9	48.4	48.5	48.0	49.1	48.4
**Age**								
18-44	50.8	45.5	44.7	45.6	41.6	39.3	38.8	43.8
45+	49.2	54.5	55.3	54.4	58.4	60.7	61.2	56.2
**Employment Status**								
Yes	62.3	62.8	63.7	66.5	65.8	64.6	62.8	64.1
No	37.7	37.2	36.3	33.5	34.2	35.4	37.2	35.9
**Years of Education**								
0-12 Years	59.1	57.3	59.6	57.9	57.1	61.2	55.5	58.2
13+ Years	40.9	42.7	40.4	42.1	42.9	38.8	44.5	41.8
**Smoking Status**								
Yes	20.9	22.7	19.5	21.9	20.1	17.2	17.2	19.9
No	79.1	77.3	80.5	78.1	79.9	82.8	82.8	80.1
**BMI**								
Healthy Weight	42.1	43.8	44.6	40.5	37.7	37.7	37.7	40.6
Overweight or Obese	57.9	56.2	55.4	59.5	62.3	62.3	62.3	59.4
**Physical Activity**								
Insufficient Physical Activity (I-MVPA)	56.9	53.4	54.4	53.5	51.6	60.3	46.8	53.8
Sufficient Physical Activity (S-MVPA)	43.1	46.6	45.6	46.5	48.4	39.7	53.2	46.2
**Screen Time**								
Low Screen Time (LST)	59.2	58.5	58.8	54.7	52.4	55.1	54.0	56.0
High Screen Time (HST)	40.8	41.5	42.2	45.3	47.6	44.9	46.0	44.0
**Combined Behaviors**								
S-MVPA/LST	25.7	27.6	27.0	26.7	24.9	21.5	29.4	26.1
I-MVPA/LST	33.5	31.0	30.9	28.0	27.5	33.6	24.6	29.8
S-MVPA/HST	17.4	19.0	18.6	19.8	23.4	18.2	23.8	20.0
I-MVPA/HST	23.4	22.5	23.6	25.5	24.1	26.7	22.2	24.0

### Measures

All surveys included items that assessed socio-demographic details of respondents including, gender, age, household income, employment status, education and smoking status. Self-reported height and body weight were used to determine BMI and classify participants into healthy weight (BMI ≤24.9) and overweight or obese (BMI ≥25.0) categories. Using the Active Australia Questionnaire all participants were asked to report the frequency and duration physical activity during recreational and transport walking, moderate and vigorous intensity physical activity in the previous week [24]. This instrument has demonstrated acceptable test-retest reliability and validity [25,26]. Engagement in sufficient MVPA (S-MVPA) was classified as achieving a minimum of 150 minutes of MVPA in at least five sessions in the previous week, participants not satisfying this criterion were classified as insufficiently physically active (I-MVPA). This method of classification is in accordance with previously described methods to reflect compliance with National Physical Activity Guidelines for Australian Adults [2,25,27]. As a marker of leisure time sedentary behavior, TV viewing is more consistently associated with overweight and obesity in females than in males [28-31]. The differences in associations between genders may be because TV viewing is not representative of other leisure time sedentary behaviors in males [32]. Thus, the current study used two measures of screen based activities as indicators of sedentary behavior, TV viewing and computer use, to better represent broader sedentary behaviors of both males and females. Duration of screen based activity reported in hours and minutes was assessed using two separate items, “What do you estimate was the total time that you spent watching TV in the last week?” and “What do you estimate was the total time you spent working in front of a computer screen in the last week?” Data from these two items was summed to provide an overall measure of screen time in the previous week and dichotomized at 21 hours into high screen time (HST) and low screen time (LST) [33]. This classification was selected a priori as it approximates an apparent threshold of screen based activity that is associated with greater risks of ill health compared to lower volumes [14,34]. No psychometric data is available on the items used to assess screen time. Similar to previous research [21], participants were further classified into four mutually exclusive groups to facilitate analyses that combines high and low levels for both physical activity and screen time: S-MVPA/LST, I-MVPA/LST, S-MVPA/HST, I-MVPA/HST (these variables are also referred to as ‘combined behaviors’).

Prior to analysis any duplicate telephone numbers between surveys were excluded. Analysis is delimited to those with complete data for all outcome variables, including screen time, resulting in a different sample size compared to previous analysis of this dataset [5]. Using data pooled from seven cross-sectional surveys (2002–2008) three separate binary logistic regression models were used to assess associations between the three different behaviors (MVPA, screen time, combined behaviors) and the likelihood to be classified as overweight or obese (Aim 1). To examine temporal trends in the prevalence of overweight or obesity, S-MVPA, and HST over the study period, separate binary logistic regression models were conducted including the ordinal variable, year of survey, as a continuous predictor. Multinomial logistic regression was used to model temporal trends in combined behaviors over the study period using S-MVPA/LST as the reference category and including year of survey as an ordinal variable. This allowed trends over the study period to be examined rather than contrasts between specific years in the study period (Aim 2). For the final analyses, the sample was stratified by each behavior group and separate binary logistic regression models were used to model the trend (year of survey) in overweight or obesity over the study period (Aim 3). All analyses were adjusted for socio-demographic variables listed in table foots; all analyses were repeated stratifying the sample by gender due to the significant interaction effect observed in the relationship between overweight or obesity and gender and combined behaviors (p<0.05).

## Results

The proportion of the overall sample classified as overweight or obese, engaging in S-MVPA and HST as separate behaviors in the pooled sample was 59.4%, 46.2% and 43.1% respectively. Examination of combined activity behaviors indicates that proportions of the overall sample classified as S-MVPA/LST, I-MVPA/LST, S-MVPA/HST, I-MVPA/HST was 26.1%, 29.8%, 20.0% and 24.0% respectively (Table 1).

Table 2 displays relationships between engagement in various activity behaviors and the likelihood to be classified as overweight or obese. Engagement in S-MVPA was inversely associated with risk of overweight or obesity in the overall sample (OR=0.85, 95% CI. 0.78-0.93) and in females (OR=0.76, 95% CI. 0.67-0.86). Engagement in HST was positively associated with risk of overweight or obesity in the overall sample (OR=1.38, 95% CI. 1.26-1.52), males (OR=1.41, 95% CI. 1.23-1.61) and in females (OR=1.40, 95% CI. 1.23-1.59). Examination of combined activity behaviors indicates that engagement in HST is positively associated with risk of overweight or obesity regardless of activity classification, and that the magnitude of association was greatest for those in the I-MVPA/HST category. This pattern of association was present in the overall sample, males and females (Table 2). Engagement in I-MVPA/LST was significantly associated with risk of overweight or obesity however this was observed only in females.

**Table 2:** Associations between physical activity, screen time, combined behavior categories and the likelihood to be classified as overweight or obese

	**Overall Sample**		**Males**		**Females**	
	**OR (95% CI)**	**p**	**OR (95% CI)**	**p**	**OR (95% CI)**	**p**
**Physical Activity**						
Insufficient Physical Activity (I-MVPA)	Reference^a^		Reference^b^		Reference^c^	
Sufficient Physical Activity (S-MVPA)	0.85 (0.78-0.93)	0.001	0.99 (0.87-1.13)	0.852	0.76 (0.67-0.86)	<0.001
**Screen Activity**						
Low Screen Time (LST)	Reference^a^		Reference^b^		Reference^c^	
High Screen Time (HST)	1.38 (1.26-1.52)	<0.001	1.41 (1.23-1.61)	<0.001	1.40 (1.23-1.59)	<0.001
**Combined Behavior**^**c**^						
S-MVPA/LST	Reference^a^		Reference^b^		Reference^c^	
I-MVPA/LST	1.10 (0.98-1.24)	0.111	0.99 (0.84-1.17)	0.912	1.20 (1.01-1.42)	0.032
S-MVPA/HST	1.29 (1.13-1.47)	<0.001	1.37 (1.13-1.66)	0.001	1.23 (1.02-1.49)	0.028
I-MVPA/HST	1.62 (1.42-1.84)	<0.001	1.42 (1.18-1.71)	<0.001	1.83 (1.53-2.19)	<0.001

^a^ Adjusted for Gender, Age, Education, Employment Status, Year of Survey, Smoking Status. n = 7908.

^b^ Adjusted for Age, Education, Employment Status, Year of Survey, Smoking Status. n = 4079.

^c^ Adjusted for Age, Education, Employment Status, Year of Survey, Smoking Status. n = 3829.

**Figure 1:**
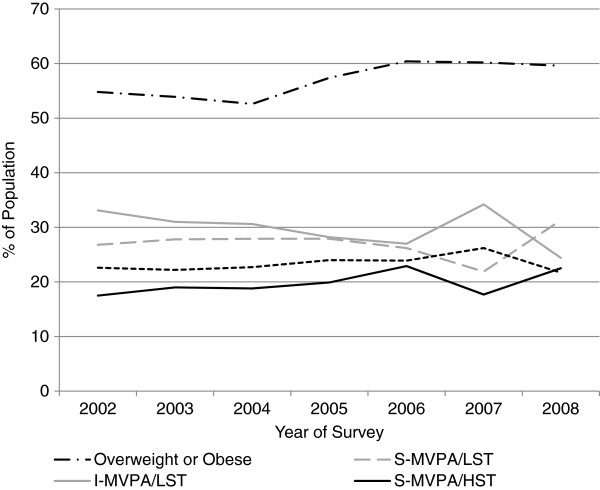
Trends in the prevalence of overweight and obesity and combined behaviors during the period 2002–2008.

Table 3 displays that the proportion of the overall population, males and females classified as overweight or obese significantly increased at the same rate over the study period. The proportion of the overall sample and females engaging in S-MVPA (OR=1.03, 95% CI. 1.01-1.05; OR=1.04, 95% CI. 1.01-1.07) also significantly increased. The proportion of the overall sample, males and females engaging in HST increased significantly over the study period (OR=1.03, 95% CI. 1.01-1.06; OR=1.04, 95% CI.1.01-1.08; OR=1.03, 95% CI. 1.00-1.07). The proportion of the overall and female population classified as I-MVPA/LST declined over the study period (Table 3). Figure 1 displays the change in the proportion of the overall population classified as overweight and obese, S-MVPA/LST, I-MVPA/LST, S-MVPA/HST and S-MVPA/LST by year of survey.

**Table 3:** Trends in overweight and obesity, physical activity, screen time and combined behaviors during the period 2002-2008

	**Overall Sample**		**Males**		**Females**	
	**OR (95% CI)**	**p**	**OR (95% CI)**	**p**	**OR (95% CI)**	**p**
**Body Weight**						
Healthy Weight	Reference^a^		Reference^d^		Reference^f^	
Overweight or Obese	1.04 (1.02-1.06)	0.001	1.04 (1.01-1.07)	0.021	1.04 (1.01-1.07)	0.015
**Physical Activity**						
Insufficient Physical Activity (I-MVPA)	Reference^b^		Reference^e^		Reference^h^	
Sufficient Physical Activity (S-MVPA)	1.03 (1.01-1.05)	0.010	1.02 (0.99-1.05)	0.211	1.04 (1.01-1.07)	0.013
**Screen Activity**						
Low Screen Time (LST)	Reference^b^		Reference^e^		Reference^h^	
High Screen Time (HST)	1.03 (1.01-1.06)	0.004	1.04 (1.01-1.08)	0.009	1.03 (1.00-1.07)	0.049
**Combined Behavior**						
S-MVPA/LST	Reference^c^		Reference^g^		Reference^i^	
I-MVPA/LST	0.97 (0.94-0.99)	0.019	0.98 (0.94-1.02)	0.327	0.95 (0.92-0.99)	0.019
S-MVPA/HST	1.03 (1.00-1.06)	0.098	1.04 (1.00-1.09)	0.073	1.02 (0.98-1.07)	0.348
I-MVPA/HST	1.00 (0.97-1.04)	0.782	1.02 (0.98-1.07)	0.334	0.99 (0.95-1.04)	0.763

^a^ Adjusted for Gender, Age, Education, Employment Status, Physical Activity, Smoking Status & Screen Time. n=7908.

^b^ Adjusted for Gender, Age, Education, Employment Status, & Smoking Status. n=7908.

^c^ Adjusted for Gender, Age, Education, Employment Status, & Smoking Status. n=7908.

^d^ Adjusted for Age, Education, Employment Status, Smoking Status, Physical Activity, & Screen Time. n=4079.

^e^ Adjusted for Age, Education, Employment Status, Smoking Status. n=4079.

^f^ Adjusted for Age, Education, Employment Status, Smoking Status, Physical Activity, & Screen Time. n=3829.

^g^ Reference Category is all other behavior categories. Adjusted for Age, Education, Employment Status, Smoking Status. n=4079.

^h^ Adjusted for Age, Education, Employment Status, Smoking Status. n=3829.

^i^ Reference Category is all other behavior categories. Adjusted for Age, Education, Employment Status, Smoking Status. n=3829.

Table 4 displays changes in the prevalence of overweight or obesity within each behavior category during the study period. Tests of interaction effects between each behaviour category and year of survey were not statistically significant for any of the outcomes presented in Table 4 (p>0.05). In the overall sample the prevalence of overweight or obesity significantly increased in those people classified as engaging in I-MVPA and S-MVPA (OR=1.05, 95% CI. 1.02-1.08; OR=1.04, 95% CI. 1.00-1.07), LST and HST (OR=1.03, 95% CI. 1.00-1.06; OR=1.05, 95% CI. 1.02-1.09) and I-MVPA/HST (OR=1.06, 95% CI. 1.01-1.11). In males the prevalence of overweight and obesity significantly increased in those classified as engaging in S-MVPA (OR=1.06, 95% CI. 1.01-1.10) and LST (OR=1.04, 95% CI. 1.00-1.09). The prevalence of overweight or obesity significantly increased in those females classified as engaging in I-MVPA (OR=1.07, 95% CI. 1.02-1.11), HST (OR=1.07, 95% CI. 1.02-1.12) and I-MVPA/HST (OR=1.10, 95% CI. 1.03-1.17).

**Table 4:** Trends in overweight and obesity within behavior categories during the period 2002-2008

	**Trend for Overweight & Obese within Behavior Categories**								
	**Overall Sample**			**Males**			**Females**		
	**n**	**OR (95% CI)**	**p**	**n**	**OR (95% CI)**	**p**	**n**	**OR (95% CI)**	**p**
**Physical Activity**									
Insufficient Physical Activity (I-MVPA)	4256	1.05 (1.02-1.08)^a^	0.002	2173	1.03 (0.99-1.08)^b^	0.154	2083	1.07 (1.02-1.11)^b^	0.007
Sufficient Physical Activity (S-MVPA)	3652	1.04 (1.00-1.07)^a^	0.039	1906	1.05 (1.00-1.10)^b^	0.032	1746	1.02 (0.97-1.06)^b^	0.319
**Screen Activity**									
Low Screen Time (LST)	4426	1.03 (1.00-1.06)^a^	0.049	2260	1.04 (1.00-1.09)^b^	0.043	2166	1.01 (0.97-1.06)^b^	0.517
High Screen Time (HST)	3482	1.05 (1.02-1.09)^a^	0.005	1819	1.03 (0.98-1.09)^b^	0.232	1663	1.07 (1.02-1.12)^b^	0.008
**Combined Behavior**									
S-MVPA/LST	2067	1.02 (0.98-1.07)^a^	0.264	1057	1.05 (0.98-1.11)^b^	0.156	1010	1.00 (0.94-1.06)^b^	0.957
I-MVPA/LST	2359	1.04 (1.00-1.08)^a^	0.070	1203	1.05 (0.99-1.11)^b^	0.131	1156	1.03 (0.97-1.09)^b^	0.302
S-MVPA/HST	1585	1.05 (0.99-1.10)^a^	0.089	849	1.06 (0.98-1.14)^b^	0.127	736	1.03 (0.96-1.11)^b^	0.354
I-MVPA/HST	1897	1.06 (1.01-1.11)^a^	0.021	970	1.01 (0.94-1.08)^b^	0.891	927	1.10 (1.03-1.17)^b^	0.005

^a^ Adjusted for Gender, Age, Education, Employment Status & Smoking Status.

^b^ Adjusted for Age, Education, Employment Status & Smoking Status.

## Discussion

This study examined relationships between physical activity, screen time, the combination of these behaviors and the likelihood to be classified as overweight or obese and also temporal trends in these outcomes during the period 2002–2008. In the overall sample and in females, engagement in S-MVPA was associated with a reduced likelihood to be classified as overweight or obese, while engagement in HST was associated with an increased likelihood to be classified as overweight or obese irrespective of activity level measured by the Active Australia Questionnaire. This pattern of associations is consistent with previous research that examined similar behavioral and health outcomes [14,21]. In males, with the exception of participation in S-MVPA, associations between screen time and combined behaviors and the likelihood to be classified as overweight or obese followed expected patterns [16,31]. The lack of association between S-MVPA and overweight or obesity in males in the current study is both in agreement [35,36], and in contrast to previous studies [37,38], and may be attributed to an positive energy imbalance, caused by energy intake exceeding energy expenditure even when males engage in S-MVPA [7,11]. The current study does not include a measure of energy intake which limits the ability to examine this mechanism.

While TV viewing is more broadly reflective of females sedentary activity than males, we attempted to offset this in the current study by incorporating a measure of computer use which is an activity that contributes more to males overall sedentary time than females [32]. Despite this, in females, associations between MVPA, screen behaviors and overweight or obesity were observed in all categories of combined behavior (I-MVPA/LST; S-MVPA/HST; I-MVPA/HST) whilst in males, associations between combined behavior and overweight or obesity were observed for selected behaviors (S-MVPA/HST; I-MVPA/HST). Several potential mechanisms may be attributed to this. The screen time and physical activity measures used in this study may better capture overall energy expenditure in females compared to males. Alternatively differences in dietary patterns may also contribute to the differing associations observed. Thus future studies should consider using measures of physical activity and sedentary behavior that capture activity in all domains and incorporate a measure of energy intake or dietary quality. Withstanding these comments, the increased risk of overweight or obesity in individuals who engaged in HST (irrespective of measured MVPA) highlights the need for interventions to target reductions in sedentary activities and increases in light, moderate and vigorous intensity physical activity to maximize overall increases in energy expenditure to reduce the risk of overweight or obesity. Increasing light intensity physical activity may be important as it is likely that this is the behavior engaged in when sitting time is reduced [39] and increased light intensity physical activity is associated with improved metabolic health [40].

Several studies have reported that the proportion of the population engaging in S-MVPA has remained stable or increased in Australian populations [4], including this population [5], and populations from other countries [3,41]. As such the more unique aspect of this study, withstanding variations between individual years examined (Table 1), is that it demonstrates that in the overall population and in females, the proportion of the population classified as overweight or obese has increased over the same time period and at a similar rate to the increased proportion of the population engaging in sufficient MVPA and high screen time. Similar patterns of change were observed in males however the change in the prevalence of S-MVPA over the study period was not statistically significant. Therefore the promising changes in the prevalence of sufficient MVPA should be viewed with cautious optimism. As it appears that some segments of the population have responded to recent efforts to promote engagement in MVPA, and at the same time also appear to spend increasing amounts of time in sedentary activities. Still, these data provide much needed information on the trends of these multiple behaviors at a population level.

The proportion of the population classified as overweight or obese increased in most physical activity and screen time groups when examined as separate behavior groups, and significantly increased only in the I-MVPA/HST group (overall sample and females) when combined behavior groups were examined. Furthermore, the rate of change in the combined activity group was marginally larger than observed when separate behavior groups were examined, although this was not significant in males. Although these conclusions must be interpreted with caution as the data are not longitudinal, and there are inconsistent data surrounding the associations between screen time and weight status compared to weight gain [15,42].

Although interesting, findings in the current study are subject to several limitations, including the use of self-report measures of MVPA, screen time and BMI. Self-reported BMI although practical in population based studies such as the current study has well acknowledged limitations [43] therefore future studies are encouraged to use objective measures of body composition to confirm the pattern of results observed in the current study. Also the measures of screen time used may be not representative of the broader time spent in sedentary activities [32]. Other limitations include the absence of longitudinal data, a lack of data on actual sedentary behavior and reliance on proxy measures of these behaviors, the absence of a measure of sedentary activity in transport related activities and the absence of a measure of energy intake. Strengths of the study are the use of consistent methodology and survey instruments over the study period and the study sample size. The sample size of the study meant that even relatively small shifts in the population prevalence of behaviors, overweight and obesity over the study period were statistically significant. However, the results do highlight important changes in behaviors at the population level.

## Conclusions

The findings of this study support previous observations that high levels of time spent engaged in screen based activity is associated with overweight or obesity in cross sectional analyses, even when MVPA is considered [16,21]. It was also observed that the prevalence of overweight or obesity appears to have increased at a similar magnitude to the prevalence of the population that engage in sufficient MVPA and high levels of screen time. Finally, we found that in the overall sample and females, the prevalence of overweight or obesity increased over time only in those who participated in insufficient levels of MVPA and high levels of screen time. Greater understanding of these relationships and trends over time requires measures of actual sedentary behaviors, such as sitting, that accurately capture the behaviors of both males and females and examination of these behaviors in both population and cohort based samples.

## Competing interests

The author declare that they have no competing interests.

## Authors’ contributions

MJD proposed the study concept, conducted statistical analyses and drafted the original manuscript. CH assisted in statistical analyses. CC, CV and WKM provided important intellectual feedback and critique of the study concept and approach. All authors contributed to the drafting and editing of the manuscript and approved the final manuscript.

## Pre-publication history

The pre-publication history for this paper can be accessed here:

http://www.biomedcentral.com/1471-2458/12/1060/prepub
